# Abdominal emergency surgery in patients with hematological malignancies: a retrospective single-center analysis

**DOI:** 10.1186/s13017-023-00481-z

**Published:** 2023-02-06

**Authors:** Philipp H. von Kroge, Anna Duprée, Oliver Mann, Jakob R. Izbicki, Jonas Wagner, Paymon Ahmadi, Sören Weidemann, Raissa Adjallé, Nicolaus Kröger, Carsten Bokemeyer, Walter Fiedler, Franziska Modemann, Susanne Ghandili

**Affiliations:** 1 Department of General, Visceral and Thoracic Surgery, University Medical Center Hamburg-Eppendorf, Martinistraße 52, 20246 Hamburg, Germany; 2 Department of Medical Psychology, University Medical Center Hamburg-Eppendorf, Martinistraße 52, 20246 Hamburg, Germany; 3 Institute of Pathology, University Medical Center Hamburg-Eppendorf, Martinistraße 52, 20246 Hamburg, Germany; 4 Department of Stem Cell Transplantation, University Medical Center Hamburg-Eppendorf, Martinistraße 52, 20246 Hamburg, Germany; 5 Department of Oncology, Hematology and Bone Marrow Transplantation with Section Pneumology, University Cancer Center Hamburg, University Medical Center Hamburg-Eppendorf, Martinistraße 52, 20246 Hamburg, Germany; 6 Mildred Scheel Cancer Career Center, University Cancer Center Hamburg, University Medical Center Hamburg-Eppendorf, Martinistraße 52, 20246 Hamburg, Germany

**Keywords:** Intestinal perforation, Intestinal obstruction, Acute cholecystitis, Anastomotic leakage, Hematological malignancies

## Abstract

**Background:**

Hematologic patients requiring abdominal emergency surgery are considered to be a high-risk population based on disease- and treatment-related immunosuppression. However, the optimal surgical therapy and perioperative management of patients with abdominal emergency surgery in patients with coexisting hematological malignancies remain unclear.

**Methods:**

We here report a single-center retrospective analysis aimed to investigate the impact of abdominal emergency surgery due to clinically suspected gastrointestinal perforation (group A), intestinal obstruction (group B), or acute cholecystitis (group C) on mortality and morbidity of patients with coexisting hematological malignancies. All patients included in this retrospective single-center study were identified by screening for the ICD 10 diagnostic codes for gastrointestinal perforation, intestinal obstruction, and ischemia and acute cholecystitis. In addition, a keyword search was performed in the database of all pathology reports in the given time frame.

**Results:**

A total of 56 patients were included in this study. Gastrointestinal perforation and intestinal obstruction occurred in 26 and 13 patients, respectively. Of those, 21 patients received a primary gastrointestinal anastomosis, and anastomotic leakage (AL) occurred in 33.3% and resulted in an AL-related 30-day mortality rate of 80%. The only factor associated with higher rates of AL was sepsis before surgery. In patients with suspected acute cholecystitis, postoperative bleeding events requiring abdominal packing occurred in three patients and lead to overall perioperative morbidity of 17.6% and surgery-related 30-day mortality of 5.9%.

**Conclusion:**

In patients with known or suspected hematologic malignancies who require emergency abdominal surgery due to gastrointestinal perforation or intestinal obstruction, a temporary or permanent stoma might be preferred to a primary intestinal anastomosis.

## Background

Gastrointestinal perforation, intestinal obstruction, and acute cholecystitis are each common indications for abdominal emergency surgery and are part of the standard repertoire of general surgery worldwide [1]. Particularly, gastrointestinal perforation and intestinal obstruction are potentially life-threatening conditions associated with high perioperative morbidity and overall mortality rates of 10% for small bowel obstruction, 5–20% for large bowel obstruction, 30% for intestinal perforation, and up to 70% for intestinal perforation with diffuse peritonitis [1–4]. In contrast, both conventional and minimally invasive cholecystectomy is standardized safe procedure associated with low perioperative morbidity and mortality [5–7].

Patients with underlying hematological diseases requiring abdominal emergency surgery are often considered to be a high-risk population due to various factors such as the urgent need for systemic treatment, particularly corticosteroids, spontaneous or therapy-related tumor necrosis, and disease- or chemotherapy-related neutropenia or thrombocytopenia [8, 9]. Thus, the optimal surgical therapy and perioperative management of gastrointestinal perforation, intestinal obstruction, and acute cholecystitis in patients with newly diagnosed or refractory or relapsed hematological malignancies remain unclear [10, 11]. Even the joined guidelines of the World Society of Emergency Surgery (WSES), Surgical Infection Society Europe (SIS-E), World Surgical Infection Society (WSIS), American Association for the Surgery of Trauma (AAST), and Global Alliance for Infection in Surgery (GAIS) about the management of acute abdomen in immunocompromised patients do not provide detailed information regarding the impact of hematological malignancies during abdominal emergency surgery [12]. Moreover, the influence of intestinal involvement of hematological malignancies in the setting of gastrointestinal perforations and intestinal obstruction as well as the impact of different systemic treatment approaches such as CD20-directed monoclonal antibody treatment, chemotherapy, and particularly intensive chemotherapy followed by autologous or allogeneic stem cell transplantation on perioperative morbidity and mortality in emergency abdominal surgical procedures is unknown. Therefore, we here report on a single-center retrospective analysis to investigate the prognostic impact of abdominal emergency surgery in patients with hematological malignancies under active treatment.

## Methods

### Study design and population

This single-center retrospective study included patients who met all the following criteria:

1. Age ≥ 18 years
2. Abdominal emergency surgery for intestinal perforation, intestinal obstruction, and/or acute cholecystitis and
3. A coexisting active hematological malignancy requiring systemic cancer treatment.

Patients with pre- or coexisting hematological malignancies under active surveillance or in posttreatment follow-up were not included in the retrospective analysis.

### Clinical data collection

All patients included in this study were identified by screening for the ICD 10 diagnostic codes (International Statistical Classification of Diseases and Related Health Problems) for gastrointestinal perforation (K63.1, K63., K25., and K31.), intestinal obstruction, and ischemia (K56. and K55.0) and acute cholecystitis (K81.). In addition, a keyword search was performed in the database of all pathology reports in the given time frame. In particular, the keywords *gastrointestinal*, *esophagus*, *stomach*, *small intestine*, *colon*, and *rectum* in combination with *lymphoma* were used.

All patients included in this study were treated at the University Medical Center Hamburg-Eppendorf, Germany, between January 2010 and May 2022. Clinical data regarding treatment and disease characterization were collected from the patient’s electronic medical records. For cytopenia assessment, the CTCAE version 5 was used [13]. The data cutoff was on May 2022. The data collection was performed in accordance with local legal requirements and was reviewed and approved by the ethics committee of the Medical Council of Hamburg (Nr. 2022-300144-WF). Informed consent was waived by the ethics committee since only anonymous data were analyzed and published.

### Endpoints

The primary aim of this study was to investigate the impact of intestinal perforations, intestinal obstructions, and/or acute cholecystitis and consecutive abdominal emergency surgery in patients with active hematological malignancies by analyzing the surgery-related 30-day and 90-day mortalities. Secondary aims were to investigate perioperative morbidities defined as anastomotic leakage (AL), fecal peritonitis, impaired wound healing, intraabdominal abscess, abdominal compartment syndrome, mesenteric ischemia, and bleeding events.

### Statistical analysis

All statistical analyses were performed by using the Statistical Package for Social Sciences statistical software, version 27.0 (IBM Corp., Armonk, New York, USA). Continuous values are presented as median with interquartile range (IQR). Nominal variables are expressed as numbers (%) and compared by Fisher’s exact test. A two-sided *p* value < 0.05 was considered statistically significant.

## Results

**Fig. 1:**
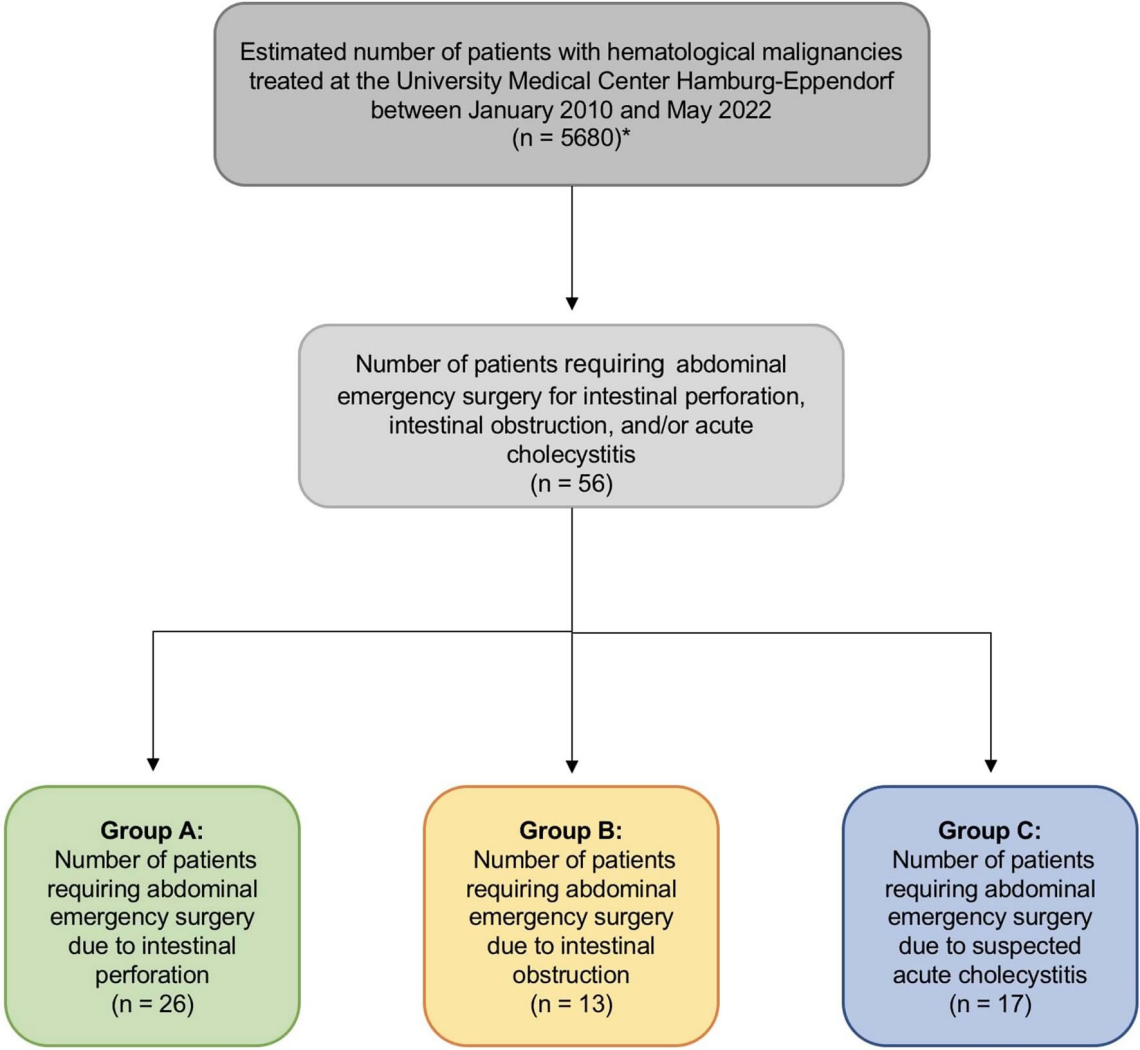
Flowchart of study design and population. Between January 2010 and May 2022, an estimated 5680 patients with hematological malignancies (including acute myeloid leukemia, myelodysplastic syndrome, multiple myeloma, acute lymphoblastic lymphoma, Hodgkin lymphoma, and non-Hodgkin lymphoma) were treated as inpatients at our department of hematology and oncology or department of stem cell transplantation. *Patients with chronic myeloid leukemia and myeloproliferative neoplasia were excluded

**Fig. 2:**
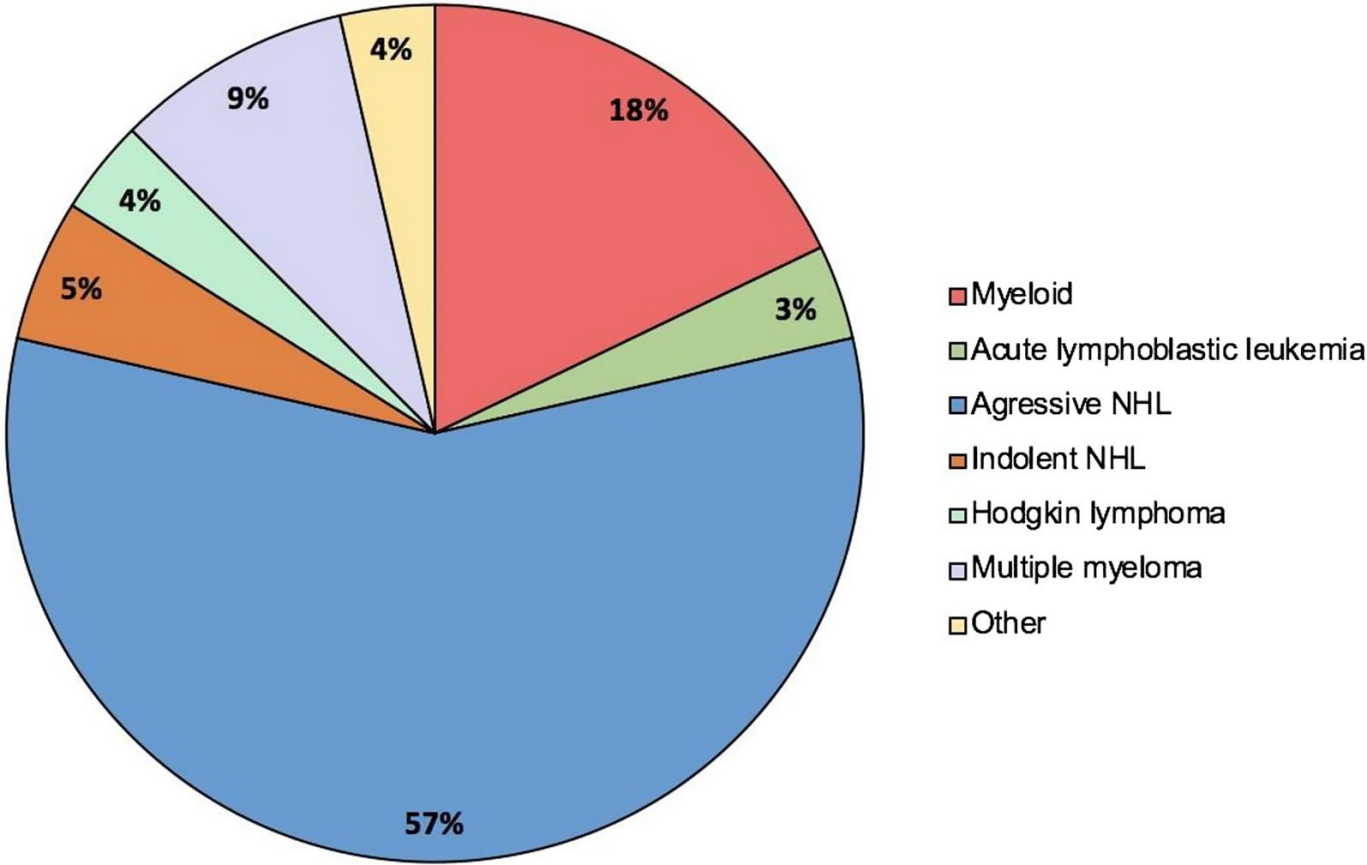
Overview of the distribution of hematological malignancies for groups A–C. NHL: Non-Hodgkin lymphoma; myeloid neoplasia consisting of acute myeloid leukemia and myelodysplastic syndrome; aggressive NHL consisting of Burkitt lymphoma, diffuse large B cell lymphoma, mantel cell lymphoma, and anaplastic large cell lymphoma; indolent NHL consisting of follicular lymphoma and T cell large granular lymphocyte leukemia; and other consisting of hemophagocytic lymphohistiocytosis

A total of 56 patients who underwent surgery for intestinal perforation, intestinal obstruction, and/or acute cholecystitis were identified and included in this analysis (Fig. 1). Patients’ demographics and characteristics are presented in Table 1 and Fig. 2.

**Table 1:** Patients’ characteristics

Total number of patients, *n*	56		
	Group A (*n* = 26)	Group B (*n* = 13)	Group C (*n* = 17)
Age at time of operation, in years, median (IQR)	62 (35.3–71.5)	64 (46–70)	58 (36–71.5)
Male sex, *n* (%)	23 (88.5)	10 (76.9)	13 (76.5%)
CCI median (range)	2 (2–5)	2 (2–5)	2 (2–5)
Distribution of hematological malignancies, *n* (%)			
Myeloid neoplasia^a^	1 (3.8)	3 (23.1)	6 (35.3)
Lymphoma^b^	23 (88.5)	5 (38.5)	11 (64.7)
Plasma cell disorders	2 (7.7)	3 (23.1)	0
Other	0	2 (15.4)	0
Disease status, *n* (%)			
Newly diagnosed	15 (57.7)	8 (61.5)	10 (58.8)
Refractory/relapsed	11 (42.3)	5 (38.5)	7 (41.2)
Intestinal or mesenteric involvement, *n* (%)	18 (69.2)	5 (38.5)	NA
Systemic treatment, *n* (%)			
CD20-directed treatment	11 (42.3)	5 (38.5)	3 (17.7)
Chemotherapy-based treatment^c^	12 (46.2)	11 (84.6)	9 (52.9)
Autologous PBSCT	1 (3.8)	0	0
Allogeneic PBSCT ≤ 100 days prior to surgery	0	3 (23.1)	4 (23.5)
Corticosteroids	11 (42.3)	6 (46.2)	5 (29.4)
Other^d^	1 (3.8)	1 (7.7)	1 (5.9)
None^e^	10 (38.5)	1 (7.7)	4 (23.5)
Median duration between last systemic treatment and surgery (IQR)^f^	21 (8–86)	23 (18–65)	29 (17–46)
Neutropenia ≥ grade 3^g^	0	4 (22.2)	2 (11.8)
Thrombocytopenia ≥ grade 3^g^	3 (11.5)	5 (38.5)	9 (52.9)
GvHD prophylaxis or treatment, *n* (%)	0	3 (23.1)	3 (17.7)
Calcineurin-inhibitors	NA	3 (23.1)	3 (17.7)
Corticosteroids	NA	1 (7.7)	1 (5.7)
Mycophenolate mofetil	NA	0	3 (17.7)
Immunoglobulin therapy	NA	2 (15.4)	3 (17.7)
Unknown	NA	0	1 (5.7)

^a^Myeloid neoplasia consisting of acute myeloid leukemia and myelodysplastic syndrome

^b^Lymphom consisting of Hodgkin lymphoma, Burkitt lymphoma, diffuse large B cell lymphoma, mantel cell lymphoma, anaplastic large cell lymphoma; follicular lymphoma and T cell large granular lymphocyte leukemia

^c^Including chemotherapy as part of the AMLSG-0909 trial (Arm B, NCT00893399), conditioning regime prior to allogeneic stem cell transplantation with busulfan or treosulfan in combination with fludarabine and with and without antithymocyte globulin and with and without total body irradiation; chemotherapy as part of the GMALL08/13 trial (NCT2881086); R-CHOP: rituximab, cyclophosphamide, doxorubicin, vincristine, and prednisolone; DHAP/DHAC: dexamethasone, cytarabine and cisplatin or carboplatin with and without rituximab; azacytidine; prednisolone and vincristine; chemotherapy analog the GMALL elderly trial (NCT00198978); ABVD: doxorubicin, bleomycin, vinblastine, dacarbazine; chemotherapy analog GMALL B-ALL/NHL 2002 (NCT00199082); LEAM: Lomustine, etoposide, cytarabine, and melphalan; rituximab and bendamustine; bortezomib and dexamethasone; daunorubicin and cytarabine; decitabine; CED: cyclophosphamide, etoposide, and dexamethasone; and chemotherapy as part of the MATRIX trial (NCT02531841)

^d^Including CAR-T cell therapy and plasmapheresis

^e^Surgical event occurred before the initiation of first-line therapy

^f^Defined as the duration between the first day of the last cycle of systemic treatment and the day of surgery

^g^According to CTCAE version 5 [13]

### Intestinal perforation (group A)

#### Patients’ characteristics

**Fig. 3:**
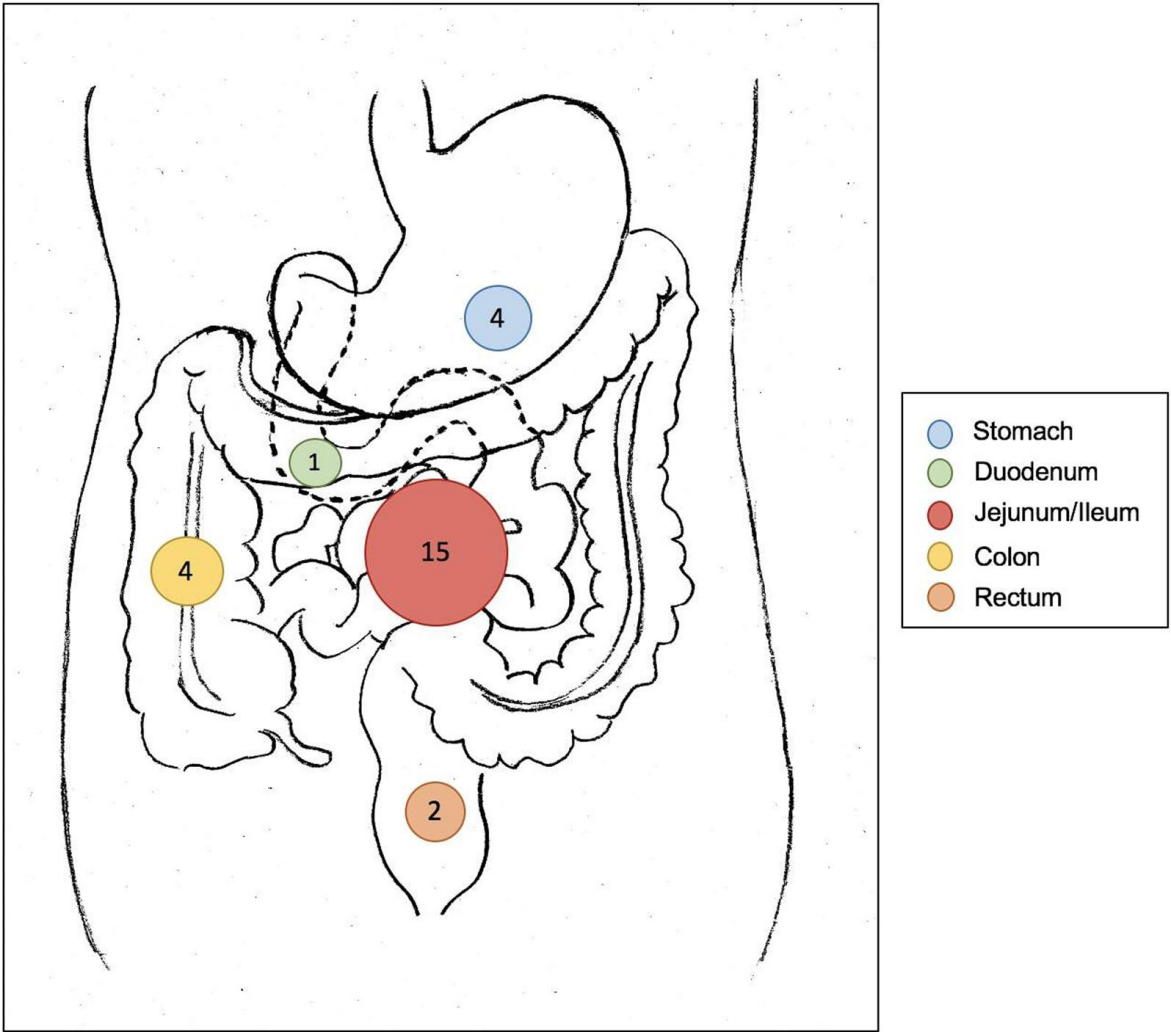
Overview of the frequency of gastrointestinal perforations depending on localization (group A, *n* = 26)

A total of 26 patients underwent surgery for intestinal perforation. The median age was 62 years (IQR 35.3–71.5), and 23 patients were male (88.5%). The most common localization of gastrointestinal perforation was jejunum or ileum in 15 patients (57.7%), followed by colon or rectum in six patients (23.1%, Fig. 3). Gastric perforation occurred in four patients (15.4%). In addition, there was one duodenal perforation. The most frequent preexisting hematological malignancy was lymphoma (88.5%), mainly consisting of diffuse large B cell lymphoma and Burkitt lymphoma. Enteral or mesenteric involvement was known in 18 patients (69.2%). At the time of abdominal emergency surgery, eleven patients (42.3%) were recently treated with CD20-directed monoclonal antibodies and 12 with chemotherapy-based regimes (46.2%). One patient (3.8%) underwent autologous stem cell transplantation. Eight patients (30.7%) had perforations in the intestinal region affected by the underlying hematological malignancy during systemic treatment.

In the majority of patients (18/26), gastrointestinal perforation occurred prior to treatment, during steroid pre-phase treatment, or during the first therapy cycle.

In group A, no patient had neutropenia > grade 2 before surgery, whereas thrombocytopenia > grade 2 occurred in three patients (11.5%). Surgical procedures for each group are presented in Table 2.

**Table 2:** Overview surgical event characteristics and surgical procedures

*Intestinal perforation (group A)*	
Localization of perforation, *n* (%)	
Stomach	4 (15.4)
Duodenum	1 (3.8)
Jejunum/Ileum	15 (57.7)
Colon	4 (15.4)
Rectum	2 (7.7)
Cause of perforation, *n* (%)	
Related to hematological malignancy	10 (38.5)
Related to systemic treatment*	8 (30.7)
Others**	8 (30.7)
Surgical procedures, *n* (%)	
Primary intestinal anastomosis^a^	17 (65.4)
Stoma^b^	8 (30.7)
Intestinal suture	1 (3.85)
*Intestinal obstruction and mesenteric ischemia (group B)*	
Localization of obstruction, *n* (%)	
Small bowel	9 (69.2)
Large bowel	4 (30.1)
Cause of obstruction, *n* (%)	
Related to hematological malignancy	5 (30.1)
Surgical procedures, *n* (%)	
Adhesiolysis without bowel resection	2 (15.4)
Bowel resection with primary anastomosis	4 (30.1)
Bowel resection with stoma	7 (53.8)
*Acute cholecystitis (group C)*	
Acalculous cholecystitis, *n* (%)	3 (17.7)
Surgical procedures, *n* (%)	
Primary open cholecystectomy	6 (35.3)
Laparoscopic cholecystectomy	11 (64.7)
Conversion	4 (36.4)

*Defined as a gastrointestinal perforation in the intestinal region affected by the underlying hematological malignancy during systemic treatment

**Including inflammation, ischemia, and ulceration

^a^Including jejunal segment resection with jejunojejunostomy, right hemicolectomy with ileotransversostomy, ileocecal resection with ileoascendostomy, gastrectomy with esophagojejunostomy, distal gastrectomy with gastrojejunostomy and jejunojejunostomy

^b^Including right hemicolectomy with terminal ileostomy, ileal resection with terminal jejunostomy, rectum exstirpation with terminal descendostomy, left hemicolectomy with terminal transversostomy, ileal segment resection with terminal ileostomy, ulcer excision and over-suturing

#### Perioperative morbidity and mortality analysis

Overall, surgery-related events occurred in eleven patients, which were predominantly AL (6/11). In all six patients, AL led to subsequent severe fecal peritonitis and septic shock requiring surgical revision with a lavage frequency of between 3 and 15 surgical interventions. AL with peritonitis and ongoing septic shock led to death in six patients. Other surgery-related events were intraabdominal abscess, impaired wound healing, necrotizing pancreatitis, bleeding, and entero-cutaneous fistula (refer to Table 3).

**Table 3:** Overview of perioperative morbidity

	In total (*n* = 56)	Group A (*n* = 26)	Group B (*n* = 13)	Group C (*n* = 17)
Number of events, *n* (%)^a^	32 (57.1)	22 (84.6)	6 (46.2)	4 (23.5)
Anastomotic leakage, *n* (%)	7 (33.3)*	6 (35.3)*	1 (25.0)*	NA
Fecal peritonitis, *n* (%)	5 (8.9)	5 (19.2)	0	NA
Impaired wound healing, *n* (%)	2 (3.6)	1 (3.8)	1 (7.7)	0
Intraabdominal abscess, *n* (%)	3 (5.4)	3 (11.5)	0	0
Abdominal compartment syndrome, *n* (%)	1 (1.8)	0	0	1 (5.9)
Mesenteric ischemia, *n* (%)	2 (3.6)	1 (3.8)	1 (7.7)	0
Bleeding, *n* (%)	9 (16.1)	3 (11.5)	3 (23)	3 (17.6)
Other, *n* (%)**	2 (3.6)	2 (7.7)	0	0
None, *n* (%)	36 (64.3)	14 (53.8)	9 (69.2)	13 (76.5)

*Of 21 (in total) with 17 patients in group A and 4 patients in group B receiving primary anastomosis

**Including necrotizing pancreatitis (*n* = 1) and entero-cutaneous fistula (*n* = 1)

^a^Partly containing multiple counts

The overall 30-day mortality observed in group A was 19.2% (5/26) with an AL-related 30-day mortality of 80% (4/5). In group A, a total of 17 patients received a primary intestinal anastomosis. 30-day mortality was significantly higher in patients with AL compared to those without AL (*p* = 0.006). A stoma was primary created in eight patients (30.7%), of which only 1 patient (12.5%) died within 30 days due to pneumogenic septic shock. In general, none-AL-related causes of death were pneumogenic septic shock and cancer progression. Overall, 90-day mortality was 34.6% (9/26), with AL-related 90-day mortality of 55.6% (5/9).

### Intestinal obstruction (group B)

#### Patients’ characteristics

A total of 13 patients underwent surgery for intestinal obstruction. However, in five cases segmental intestinal ischemia was found intraoperatively instead of the suspected diagnosis of ileus. The median age was 64 years (IQR 46–70), and ten patients were male (76.9%). Small bowel obstruction occurred in nine patients (69.2%). The most frequent preexisting hematological malignancies were lymphatic malignancies (38.5%) including four patients with previously existing enteral or mesenterial involvement. The most common cause of intestinal obstruction was tumor obstruction in five patients, followed by adhesions in two patients. At the time of abdominal emergency surgery, five patients were treated with CD20-directed monoclonal antibodies (38.5%), eleven with chemotherapy-based regimes (84.6%), and three patients underwent allogeneic stem cell transplantation ≤ 100 days before surgery (23.1%).

#### Perioperative morbidity and mortality analysis

Overall, surgery-related events were observed in six patients (46.2%). Of those, AL was seen in one patient and impaired wound healing in another patient. Intestinal ischemia occurred in one patient. Bleeding complications were observed in three patients. Overall, 30-day mortality was 46.2% (6/13) only due to pneumogenic septic shock and cancer progression without any surgery-related deaths.

#### Perioperative morbidity of primary intestinal anastomosis and primary stoma creation

A total of 21 patients from groups A and B received a primary intestinal anastomosis. Overall, the AL rate was 33.3% (7/21). Six AL occurred after small bowel reconstruction and one after esophagojejunostomy. Five patients received a stapler anastomosis, whereas hand-sewn anastomosis was performed in 13 patients (unknown in the remaining three). The anastomotic technique had no significant influence on the occurrence of AL. Sepsis before surgery was associated with higher rates of AL (*p* = 0.02). Systemic treatment before surgery was not related to an increasing rate of AL (*p* = 0.35). Furthermore, lymphatic disease, perioperative neutropenia, and thrombocytopenia, CD20-directed treatment, chemotherapy, intensive chemotherapy with autologous or allogeneic stem cell transplantation, and systemic corticosteroids were not associated with higher rates of anastomotic leakages.

Primary stoma creation was performed in a total of 15 patients in group A and B. In these patients, no surgical complications were observed.

### Acute cholecystitis (group C)

#### Patients’ characteristics

A total of 17 patients underwent surgery for suspected acute cholecystitis. The median age was 58 years (IQR 36–71.5), and 13 patients were male (76.5%). Acalculous cholecystitis was observed in three patients (17.7%). At the time of abdominal emergency surgery, three patients (17.7%) were last treated with CD20-directed monoclonal antibodies and nine with chemotherapy-based regimes (52.9%). Four patients (23.5%) underwent allogeneic stem cell transplantation. Primary open cholecystectomy was performed in six patients (35.3%) and primary laparoscopic cholecystectomy in eleven patients (64.7%). The conversion was necessary for four of eleven patients leading to a conversion rate of 36.4%. Reasons for conversion were bleeding, advanced local peritonitis, gall bladder perforation, and septic shock (each in one patient).

#### Perioperative morbidity and mortality analysis

The overall 30-day mortality observed in group C was 47.1% (8/17), with a cholecystitis-related 30-day mortality of 5.9% (1/17). Other causes of death were acute liver failure in two patients due to lymphoma progression and pneumogenic septic shock in five patients including all patients with acalculous cholecystitis. Postoperative bleeding events requiring abdominal packing occurred in three patients and lead to overall perioperative morbidity of 17.6%.

## Discussion

Hematologic patients requiring abdominal emergency surgery are usually considered to be a high-risk population based on disease and treatment-related myelosuppression and in some cases also immunosuppression due to impaired polyclonal immunoglobulin production or B cell depletion [9]. However, up to now the optimal surgical therapy and perioperative management of patients with abdominal emergency surgery in patients with coexisting hematological malignancies have not been well defined [11]. First, by investigating the clinical course of 26 patients with active hematological malignancies requiring abdominal emergency surgery due to gastrointestinal perforation (group A) we observed perioperative morbidity of 38.5% predominantly caused by gastrointestinal AL. Remarkably, the majority of AL occurred after small bowel reconstruction and led to subsequent severe fecal peritonitis, septic shock, and death which resulted in an exceptionally high 30-day mortality of 80%. Besides, the AL-related 30-day mortality observed in our study appears to be particularly high in comparison with the general rate of gastrointestinal AL of 8.4% independent of the anastomotic localization [14, 15]. Furthermore, the rate of AL reported in the subgroup of small bowel anastomoses in the setting of traumatic perforation is only 3.4% which is even more in contrast to the AL rate of 33.3% in our study population [16]. In contrast, 30-day mortality was lower in patients with primary stoma creation after gastrointestinal perforation compared to those receiving a primary anastomosis (23.5%).

Interestingly, regarding primary anastomosis in general we did not observe any negative impact of any type of systemic treatment before surgery on the development of AL. Moreover, none of these patients died from surgical complications. One of the few publications that reported on surgical treatment of acute abdominal complications in hematological patients is a retrospective single-center analysis that investigated the outcome and prognostic factors in 58 patients including 26 patients with gastrointestinal perforation. In 2017, Mokart and colleagues reported surgery-related perioperative morbidity of 26% which is noticeably lower compared to the perioperative morbidity observed in our study population. In contrast to our study, the authors observed AL in only one of 26 patients with gastrointestinal perforation and did not provide further detailed information on the number of patients receiving primary intestinal anastomoses or stomata. Furthermore, they were able to demonstrate that neither neutropenia nor thrombocytopenia did negatively impact the prognosis of hematological patients requiring surgery due to gastrointestinal perforation which parallels our results [17]. In fact, in our study population sepsis before surgery appears to be the only factor associated with the occurrence of an AL.

Moreover, regarding the localization of intestinal perforation, our results are in line with those of Vaidya et al. who investigated the incidence and clinical features of bowel perforation in 92 patients with indolent and aggressive lymphoma. The most frequently observed localization of intestinal perforation reported by Vaidya et al. was the small bowel with 58% which is paralleled by 61.5% in our patient cohort [18].

Next, we investigated the clinical course of 13 patients receiving abdominal emergency surgery due to the suspected diagnosis of intestinal obstruction (group B). In the majority of this subgroup, stoma creation was performed. Similar to our results in group A, we observed here a high rate of AL of 25% with repetitive abdominal revision surgery due to fecal peritonitis. In contrast, only one surgical revision was necessary in those patients with primary stoma formation and no patient died from surgical complications. Furthermore, by investigating the clinical course of 17 patients receiving surgery due to suspected acute cholecystitis we observed a conversion rate of 36.4% which is substantially higher than the usually reported conversion rate of 15.9%. In addition, bleeding events requiring abdominal packing in all three cases did frequently occur in our subgroup leading to overall surgical-related morbidity of 17.6% which is also increased compared to 5.9% reported by Giger et al. [7].

Due to the relatively small cohort size, the retrospective study design, and the heterogeneous study population, there is a risk of potential selection bias and residual confounding variables (e.g., surgeons’ experience) in this analysis.

## Conclusion

To the best of our knowledge, we were able to show for the first time that primary intestinal anastomosis is associated with exceptionally high rates of AL and subsequent high 30-day mortality in patients with hematological malignancies requiring abdominal emergency surgery. Based on our results, we suggest that if abdominal emergency surgery is required due to gastrointestinal perforation in patients with known or suspected hematological malignancies, temporary or permanent intestinal stoma might be preferred to primary intestinal anastomosis not only to reduce the risk of septic shock due to fecal peritonitis but also to enable treatment of the underlying disease to be continued as quickly as possible. However, as hematological malignancies represent a large cohort of different diseases with various intensive systemic therapy approaches and some surgical emergencies can occur in various ways and at different time points, there will be no way of conducting prospective or even randomized trials. Thus, we need to retrieve knowledge from large case series. While our data clearly show increased mortality and morbidity in patients with hematological malignancies requiring abdominal emergency surgery, they are also assuming that these patients can have a positive outcome of emergency surgery in most cases and thus procedures should not be withheld in these situations.

## Data Availability

The datasets used and/or analyzed during the current study are available from the corresponding author on reasonable request.

## Abbreviations

AL: Anastomotic leakage
IQR: Interquartile range
